# Establishment of a novel lysosomal signature for the diagnosis of gastric cancer with *in-vitro* and *in-situ* validation

**DOI:** 10.3389/fimmu.2023.1182277

**Published:** 2023-05-05

**Authors:** Qi Wang, Ying Liu, Zhangzuo Li, Yidan Tang, Weiguo Long, Huaiyu Xin, Xufeng Huang, Shujing Zhou, Longbin Wang, Bochuan Liang, Zhengrui Li, Min Xu

**Affiliations:** ^1^ Department of Gastroenterology, Affiliated Hospital of Jiangsu University, Jiangsu University, Zhenjiang, China; ^2^ Department of Cardiology, Sixth Medical Center, PLA General Hospital, Beijing, China; ^3^ Department of Cell Biology, School of Medicine, Jiangsu University, Zhenjiang, China; ^4^ Faculty of Medicine, University of Debrecen, Debrecen, Hungary; ^5^ Department of Pathology, Affiliated Hospital of Jiangsu University, Jiangsu University, Zhenjiang, China; ^6^ Faculty of Dentistry, University of Debrecen, Debrecen, Hungary; ^7^ Department of Clinical Veterinary Medicine, Huazhong Agricultural University, Wuhan, China; ^8^ Faculty of Chinese Medicine, Nanchang Medical College, Nanchang, China; ^9^ Department of Oral and Maxillofacial-Head and Neck Oncology, Shanghai Ninth People’s Hospital, Shanghai Jiao Tong University School of Medicine, College of Stomatology, Shanghai Jiao Tong University, Shanghai, China; ^10^ National Center for Stomatology and National Clinical Research Center for Oral Diseases, Shanghai JiaoTong University, Shanghai, China; ^11^ Shanghai Key Laboratory of Stomatology, Shanghai JiaoTong University, Shanghai, China

**Keywords:** lysosome, gastric cancer, diagnosis, machine learning, immunotherapy, chemotherapy

## Abstract

**Background:**

Gastric cancer (GC) represents a malignancy with a multi-factorial combination of genetic, environmental, and microbial factors. Targeting lysosomes presents significant potential in the treatment of numerous diseases, while lysosome-related genetic markers for early GC detection have not yet been established, despite implementing this process by assembling artificial intelligence algorithms would greatly break through its value in translational medicine, particularly for immunotherapy.

**Methods:**

To this end, this study, by utilizing the transcriptomic as well as single cell data and integrating 20 mainstream machine-learning (ML) algorithms. We optimized an AI-based predictor for GC diagnosis. Then, the reliability of the model was initially confirmed by the results of enrichment analyses currently in use. And the immunological implications of the genes comprising the predictor was explored and response of GC patients were evaluated to immunotherapy and chemotherapy. Further, we performed systematic laboratory work to evaluate the build-up of the central genes, both at the expression stage and at the functional aspect, by which we could also demonstrate the reliability of the model to guide cancer immunotherapy.

**Results:**

Eight lysosomal-related genes were selected for predictive model construction based on the inclusion of RMSE as a reference standard and RF algorithm for ranking, namely ADRB2, KCNE2, MYO7A, IFI30, LAMP3, TPP1, HPS4, and NEU4. Taking into account accuracy, precision, recall, and F1 measurements, a preliminary determination of our study was carried out by means of applying the extra tree and random forest algorithms, incorporating the ROC-AUC value as a consideration, the Extra Tree model seems to be the optimal option with the AUC value of 0.92. The superiority of diagnostic signature is also reflected in the analysis of immune features.

**Conclusion:**

In summary, this study is the first to integrate around 20 mainstream ML algorithms to construct an AI-based diagnostic predictor for gastric cancer based on lysosomal-related genes. This model will facilitate the accurate prediction of early gastric cancer incidence and the subsequent risk assessment or precise individualized immunotherapy, thus improving the survival prognosis of GC patients.

## Introduction

Given the characteristics of gastric cancer itself, which is a malignant tumor caused by a combination of genetic, site-specific, and microbiological factors, the overall prognosis of gastric cancer patients has not improved significantly (1, 2). Both the incidence and mortality rates of gastric cancer are among the highest of all malignancies today, and this situation is becoming increasingly alarming in Eastern Asia (3). Under the paradigm of accurate medicine, integrating the exploitation of multi-omics data and combining informatics technology with current traditional clinical screening tools at different levels to achieve more accurate and convenient early cancer screening is a challenge that scholars are currently committed to solving (2, 4, 5). Throughout this process, the exploration of new early diagnostic and prognostic biomarkers or preclinical diagnostic or rating models could benefit the current gastric cancer diagnosis and treatment by targeting prevention and personalized medical care.

In light of the emerging potential usefulness of lysosomes in the treatment of a wide range of malignancies and non-malignancies, such as neurological and cardiovascular diseases, targeting lysosomes presents a novel solution for the prevention of gastric cancer (6). Long thought to act as a caretaker or housekeeper in the context of the individual cell, it was mainly due to its role as a static organelle and its initially perceived function, degradation, which was not related to the altered state of the unit (7). Nevertheless, the continuing pursuit of microscopic perspectives, combined with multi-omics and bioinformatics methodologies, allows for a clearer understanding that lysosomes are far from being isolated islands from other cell organelles (8). More generally, from the understanding that it is a participant in intracellular homeostasis, lysosomes were shown to impact metabolic signaling, cell proliferation and differentiation, immune responses, and other procedures (9). Additionally, the tight association with autophagy allows it to be likewise engaged in diverse modes of cell death, such as ferroptosis, autophagy-dependent cell death, apoptosis, and pyroptosis (10, 11). Considering the crucial role of lysosomes in the progression of various human diseases as well as their prevalence, the value of lysosomes in translational medicine could likewise be maximized by integrating multi-omics data in the era of precision medicine (12).

**Figure 1:**
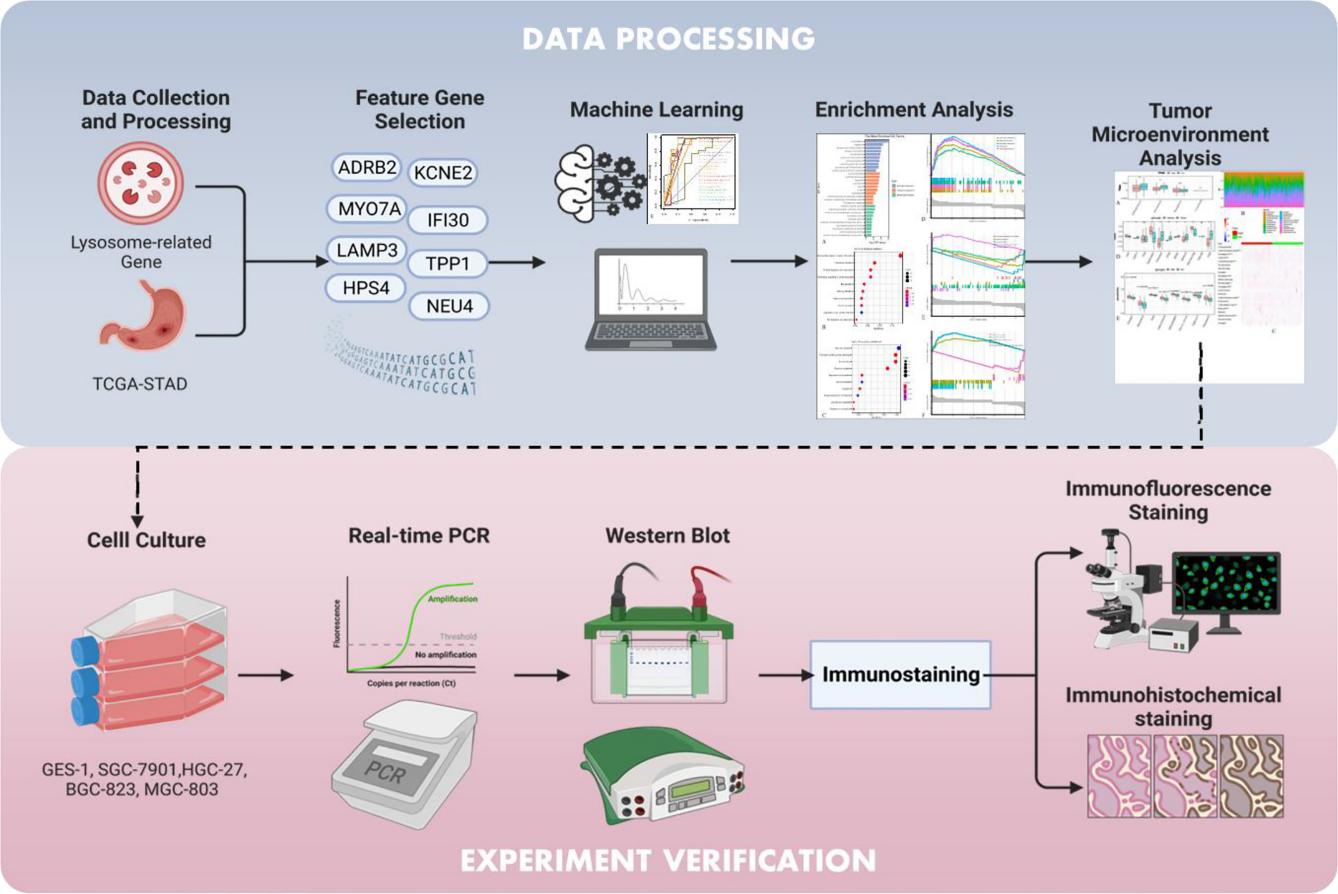
Graphical abstract of the present study.

Therefore, we attempted to integrate and compare more than 20 mainstream classifying algorithms in machine learning to identify the most ideal diagnostic model for STAD, and subsequent insights involving the tumor immune microenvironment and drug sensitivity revealed the potential immunotherapeutic applicability of our lysosomal gene model. In addition, a series of validation of the model constructed genes for IFI30, including expression validation in different dimensions and functional assays, have more or less confirmed that the model constructed genes themselves serve as negligible risk factors for GC (**Figure 1**).

## Materials and methods

### Data collection and processing

In the present study, we retrospectively collected the lysosome-related genes from the Msigdb (https://www.gsea-msigdb.org/gsea/msigdb/cards/LYSOSOME) repository and the transcriptomic data with matching clinical information of Stomach Adenocarcinoma (STAD) from the TCGA (https://www.cancer.gov/tcga) cohort (13). All the data involved in the present study were processed by R Foundation and Python software and randomly divided into the training set and the validation set in a ratio of 0.8. Notably, if not specified, a P-value<0.05 is considered statistically significant and might be annotated as * within the figures. Moreover, **, ***, and **** might appear within the figures to indicate the P-value thresholds 0.01, 0.001, and 0.0001, respectively.

### Feature gene selection prior to diagnostic model construction

We first used the Recursive Feature Elimination (REF) approach to determine the optimal number of feature genes for model construction (14). Then, we applied Random Forest (RF) algorithm to rank their significance from the highest to the lowest (15). Selected top feature genes were further utilized for model construction.

### Machine learning

According to the “No Free Lunch” theorem, we exhaustively ran out of 20 mainstream machine-learning algorithms to find the most ideal diagnostic model for STAD (16). The algorithms include Linear Regression, Ridge Regression, RidgeCV, Linear LASSO, LASSO, ElasticNet, BayesianRidge, Logistic Regression, SGD, SVM, KNN, Naive Bayes, Decision Tree, Bagging, Random Forest, Extra Tree, AdaBoost, GradientBoosting, Voting, and ANN. Their performances were mainly assessed by the diagnostic Receiver Operative Characteristic (ROC) curves in which the Area Under Curve (AUC) represented the predictive power, but we also considered other parameters for evaluation (i.e., accuracy, precision, recall, and F1 measurement) to ensure our criteria were rigorous enough. The greater the AUC value indicated the better accuracy and robustness of the model. All the aforementioned curves were created by the Python package “sklearn”.

### Learning curve

A learning curve is a graphical depiction of the connection between proficiency and experience. Through machine learning, artificial intelligence mimicked human behavior, in essence. Therefore, by visualizing its learning process in the training set and its predictive performance in the validation set, we would be able to see if the model worked robustly in a direct manner.

### Decision curve analysis

Usually, prognostic models and diagnostic tests are mathematically evaluated with measures of accuracy that do not consider clinical outcomes. To overcome this disadvantage, DCA, which is often used to compare the efficacy of different predictive models to maximize the clinical benefits when false positives and false negatives are inevitable, was introduced into the present study (17, 18).

### Single-sample gene set enrichment analysis

It is an extension of Gene Set Enrichment Analysis (GSEA) that produces distinct enrichment scores for every possible pairing of a sample and gene set (19, 20). Each ssGSEA score would show the extent to which the genes in a certain gene set are coordinately up- or down-regulated within an individual sample. In the present study, we defined the sum of the ssGSEA score in focus on the feature genes selected from the above as Lysosomal Index (LI). All the TCGA samples were allocated into high- and low-LI groups in the following bioinformatic analytics.

### Functional enrichment analysis

Traditionally, the statistical principle of enrichment analysis is to use hypergeometric distribution to test the significance of a certain functional class in a group of genes. In recent years, scientists also tended to perform such analytics at a gene set level, treating numerous genes of interest as a whole. In the present study, the functional enrichment analysis was carried out not in both ways.

### Analysis of the tumor microenvironment

The R package “ESTIMATE” was employed to calculate the scores of stromal cells, infiltrating immune cells, and tumor purity on the basis of gene expression. In this way, we unraveled the in-depth correlation between the LI and the surroundings of the malignant cells. The immune cell infiltration analysis was done by the CIBERSORT algorithm which is a widely used immunoinformatic tool to uncover the immunological implications of various diseases nowadays (21).

### Cell culturing

Human gastric mucosal epithelial cells GES-1 and human gastric cancer cell lines SGC-7901, BGC-823, MGC-803, and HGC-27, which were identified by DNA typing of STR sequences, were purchased from Shanghai Institutes of Biological Sciences (CAS). The above cells were cultured at 37°C in a humidified incubator containing 5% CO2, cultured with DMEM (HyClone) mixed with 10% fetal bovine serum (Gibco, Carlsad, CA, USA), where the culture medium was changed once a day.

### Real-time PCR

After extraction of total RNA using RNAiso Plus (Takara, Dalian, China), RT-qPCR was performed using Revertaid First Strand cDNA synthesis kit (Thermo Fisher Science, Waltham, MA, USA), which was conducted under the manufacturer’s protocol. An SYBR Green-based real-time fluorescent quantitative polymerase chain reaction was carried out using GAPDH as an internal reference, in which the primers involved were GAPDH-F: GGTGAAGGTCGGTGTGAACG; GAPDH-R: ZCTCGCTCCTGGAAGATGGTG, IFI30-F: GTGGGAGTTCAAGTGCCAGCAT; IFI30-R: GCAGACAATGGTCAGGAAGGCT. The aforementioned results were calculated by the 2-ΔΔCT method to get the relative fold change of RNA expression.

### Western blot

The target cells subjected to cold PBS wash were treated with cell lysis buffer according to the reagent manufacturer’s recommendation. And the extracted proteins were electrophoresed and separated on SDS-PAGE gels as background, and then transferred to PVDF membranes. After closed fixation with 5% bovine serum albumin, they were incubated with primary antibodies overnight at 4°C, followed by incubation with secondary antibodies.

### Immunofluorescence staining

The five cells mentioned above GES-1, SGC-7901, BGC-823, MGC-803, and HGC-27, were treated as recommended by the reagent supplier and incubated with IFI30 primary antibody, and after 12 h incubation with specific secondary antibody at 37°C in the dark for 2 h. After staining with DAPI at room temperature, fluorescent images were captured with a confocal microscope system.

### Immunohistochemical staining

Pre-fixed and paraffin-embedded pathological material was cut to 5 mm width and dewaxed. Endogenous peroxidase was inactivated with 3% H2O2. The treated slices were incubated overnight at 4°C with the corresponding protein antibodies, then incubated with secondary antibodies for 30 min followed by final chromogenic color development with freshly prepared DAB reagent.

### Transwell, invasion and wound healing assay

Transfected gastric cancer cells with high expression of IFI30 were inoculated in 6-well plates, and the cells were scratched manually to create scratches, which were placed under standard culture conditions for 48 h. Every 12 h, photographs were taken to observe the healing of the scratches. The concentration of 100,000 pretreated cells (100 μL) and the control cells were inoculated into transwell chambers, and the transwell assay and invasion assay were performed with or without matrix gel, after 24 h of cell growth, the cells were fixed with paraformaldehyde and stained with 0.05% crystalline violet for over 30 mins and then counted.

### Statistical analysis

The bioinformatics of this study involved was operated by R software as well as python software, t-test and Kruskal-wallis test were performed for the evaluation of pairwise transcriptomic data and Pearson or Spearman methods were employed for the evaluation of the correlation tests involved, p-values < 0.05 (*P < 0.05) were considered significant, for which **, p<0.01, ***, p < 0.001, and ****, p < 0.0001.

## Results

### 8 lysosome-related genes were chosen to construct the predictor

**Figure 2:**
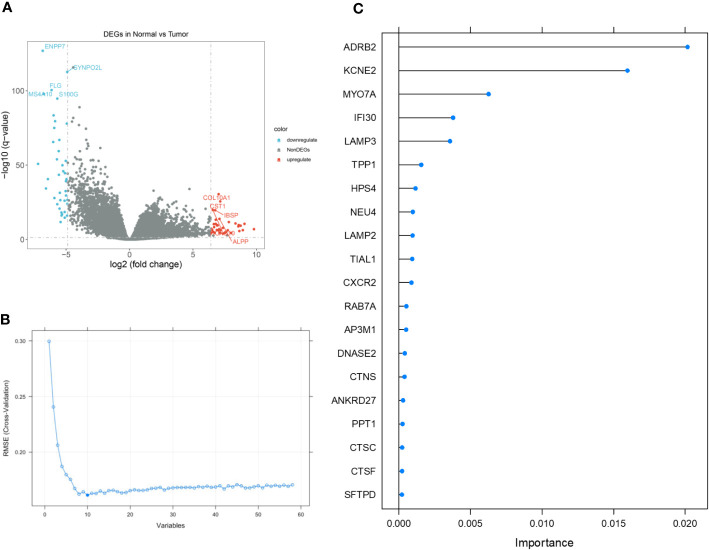
The detailed process of feature gene selection. **(A)** Volcano plot demonstrating the up- and down-regulated genes in Stomach Adenocarcinoma (STAD). **(B)** Scree plot demonstrating the change of cross-validation Root Mean Square Deviation (RMSE) with different amounts of feature genes involved in the construction of diagnostic predictor. **(C)** Importance ranking by Random Forest (RF) algorithm. The top 8 genes were selected.

**Figure 3:**
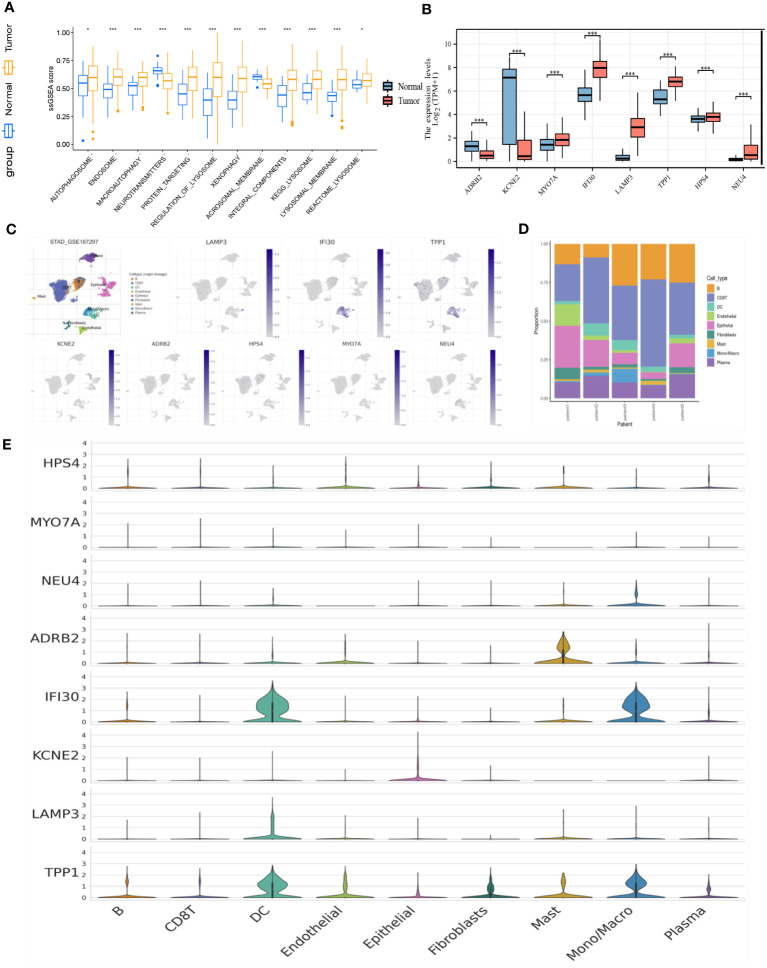
**(A)** Box plot demonstrating the enriched items with statistical significance. **(B)** Box plot demonstrating the expression of selected genes in the TCGA dataset. **(C)** Annotation of all cell types in GSE167297 and percentage of each cell type. **(D)** Illustrations of the percentage of cells in different samples. **(E)** Expression of ADRB2, KCNE2, MYO7A, IFI30, LAMP3, TPP1, HPS4, and NEU4 in diverse cells. *p< 0.05; ***, p < 0.001.

Based on the stratification of tumor samples and healthy controls, we exhaustively screened the differentially expressed genes (DEGs), within which the majority of lysosome-related genes were presented (**Figure 2A**). Then, by utilizing the Recursive Feature Elimination (REF) algorithm, it was observed that when the number of genes involved in model construction was less than 8, the Root Mean Square Deviation (RMSE) increased significantly. Meanwhile, when this number exceeded 8, the RMSE fluctuated in an acceptable range (**Figure 2B**). Therefore, it was determined that using 8 lysosome-related genes for predictor construction was the most ideal solution. To specify the top 8 candidate genes, we used Random Forest (RF) algorithm to rank their importance in Stomach Adenocarcinoma (STAD) diagnosis. As a result, ADRB2, KCNE2, MYO7A, IFI30, LAMP3, TPP1, HPS4, and NEU4 were chosen (**Figure 2C**). The inter-correlation analyses between these genes were also conducted to give references to their characterization in STAD and healthy controls (**Supplementary S1**). Additionally, we explored the difference in the enrichment of lysosome-related gene sets. Of note that lysosome and lysosomal membrane were among the most enriched items across both KEGG and Reactome databases (**Figure 3A**). The detailed expression level of each gene for the construction of the predictive model was shown in the manner of a box plot (**Figure 3B**). Aiming to gain a deeper appreciation of the above modeled genes, we probed the expression of these genes in the gastric cancer single cell dataset GSE167297 (**Figures 3C, D**). The above genes were all found to be expressed in clusters of cells, with LAMP3, IFI30, TPP1, KCNE2, ADRB2 being more significantly displayed. Among them, LAMP3, IFI30, TPP1, KCNE2 and ADRB2 were more significantly represented on functional cells such as DC cells, macrophages, endothelial cells and mast cells, suggesting more or less the reliability of the current model (**Figure 3E**).

### Extra tree was the most superior machine learning algorithm

**Figure 4:**
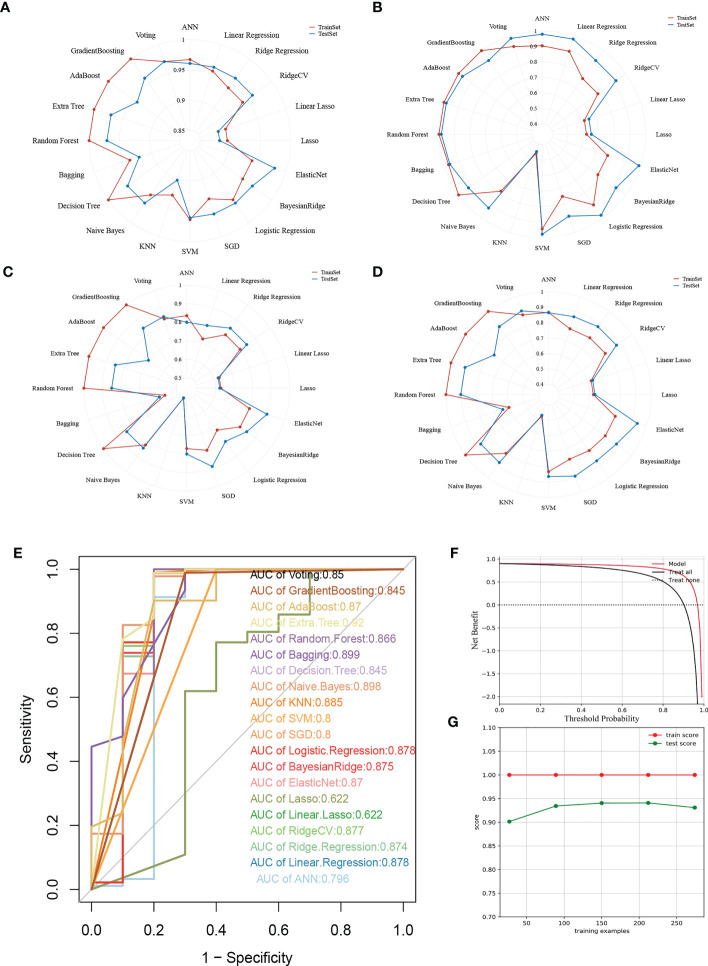
Multifaceted evaluation of 20 mainstream machine-learning models. **(A-D)** Radar plots demonstrating accuracy, recall, and F1 measurement in the training set and test set, respectively. **(E)** Receiver Operative Curve (ROC) in which the Area Under Curve (AUC) value of each machine learning model was compared. In general, an AUC value over 0.7 was thought to be a good predictive performance. **(F)** Decision Curve Analysis (DCA) for the Extra Tree model. The Guilherme position of the curve, the greater the clinical benefits. **(G)** Learning curve of the Extra Tree model. The closer the learning and testing results, the more robustness the model possesses.

To ensure a comprehensive comparison of the 20 mainstreamed machine-learning algorithms, we did not solely elucidate our models from ROC-AUC values, but in multiple aspects including accuracy, precision, recall, and F1 measurement. Regarding the accuracy, recall, and F1 measurement, Extra Tree and Random Forest were found to be the most well-performing models (**Figures 4A, C, D**), while for precision, except for Linear LASSO and LASSO, all the rest of models exerted quite satisfying predictions (**Figure 4B**). On the other hand, while all the models were holding a high ROC-AUC value of over 0.7, the general bar to consider a model was good enough in classifying questions, Extra Tree possessed a leading ROC-AUC value of up to 0.92, followed by Bagging and Naïve Bayes (**Figure 4E**). Then, we inspected the clinical benefits that the Extra Tree model could bring into real-world practice through the DCA curve. As indicated, the model offered betterment when compared with the treat-all and treat-none groups (**Figure 4F**). We also reviewed the learning process of the AI behind the model, for which it was visualized in the form of a learning curve (**Figure 4G**). Through the curve, it was observed that the learning score in the training set was stable and perfect. Overall, the difference between the training score and the testing score was less than 10%, therefore, it was deemed as a model with high generalizing ability.

### Patients with high- and low-lysosome index showed significant morphological changes in their external gastric appearance

**Figure 5:**
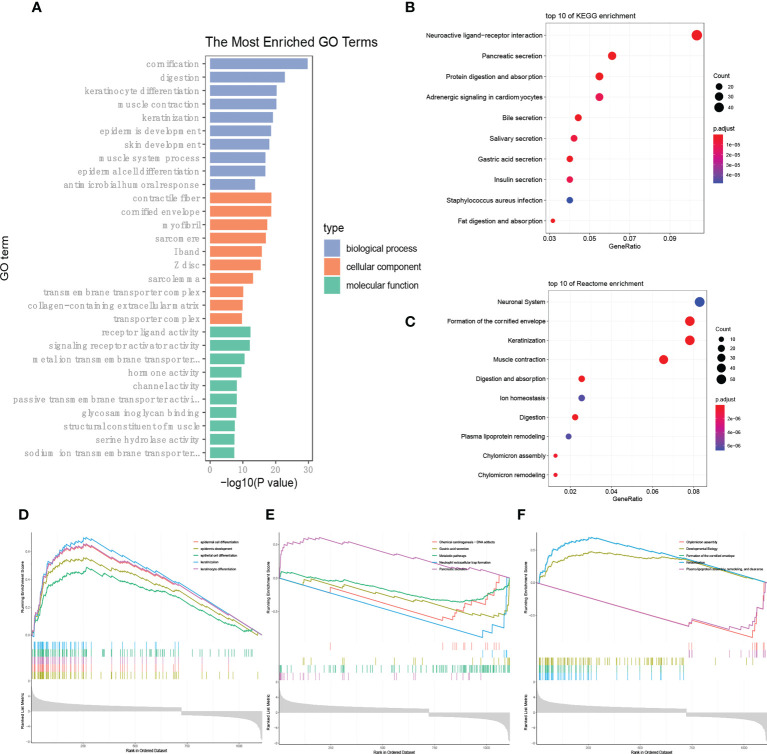
LI-based enrichment analysis. **(A-C)** Presentation of the top 10 differential pathways from GO **(A)**, KEGG **(B)**, and Reactome **(C)** enrichment analysis via the traditional method. **(D-F)** Results of GSEA analysis according to the GO **(D)**, KEGG **(E)**, and Reactome **(F)** databases, respectively.

Traditional enrichment analysis was performed to identify whether the enriched gene ontology (GO) and signaling pathways were distinguished between the high- and low-LI groups. The results according to the GO database indicated that cornification was the most distinguishable biological process, followed by digestion, keratinocyte differentiation, muscle contraction, and keratinization (**Figure 5A**). Notably, differences in cellular components including contractile fiber and cornified envelope were also prominent. In short, different LI seemingly raise morphological changes on the gastric surface. On the other hand, we found the most enriched pathways were not similar when the KEGG database and the Reactome database were applied separately. From the KEGG side, we observed that pathways relevant to secretion were outstanding, such as Pancreatic secretion, Bile secretion, Salivary secretion, Gastric acid secretion, and Insulin secretion (**Figure 5B**). However, through the Reactome database, the GO enrichment results were supported as the top pathways contained the Formation of the cornified envelope, Keratinization, Muscle contraction, and so on (**Figure 5C**). Under such circumstances, we further conducted a GSEA analysis to determine the secrets behind it. Again, changes in the epithelial morphology were seen in the GO enrichment results (**Figure 5D**), while the results of KEGG enrichment remained secretion-centered (**Figure 5E**). Moreover, consequently, Keratinization appeared again among the most enriched Reactome pathways (**Figure 5F**).

### Patients in the high- and low-LI groups possessed different tumor immunological microenvironment characteristics, predictive immunotherapy efficacy, and chemosensitivity

**Figure 6:**
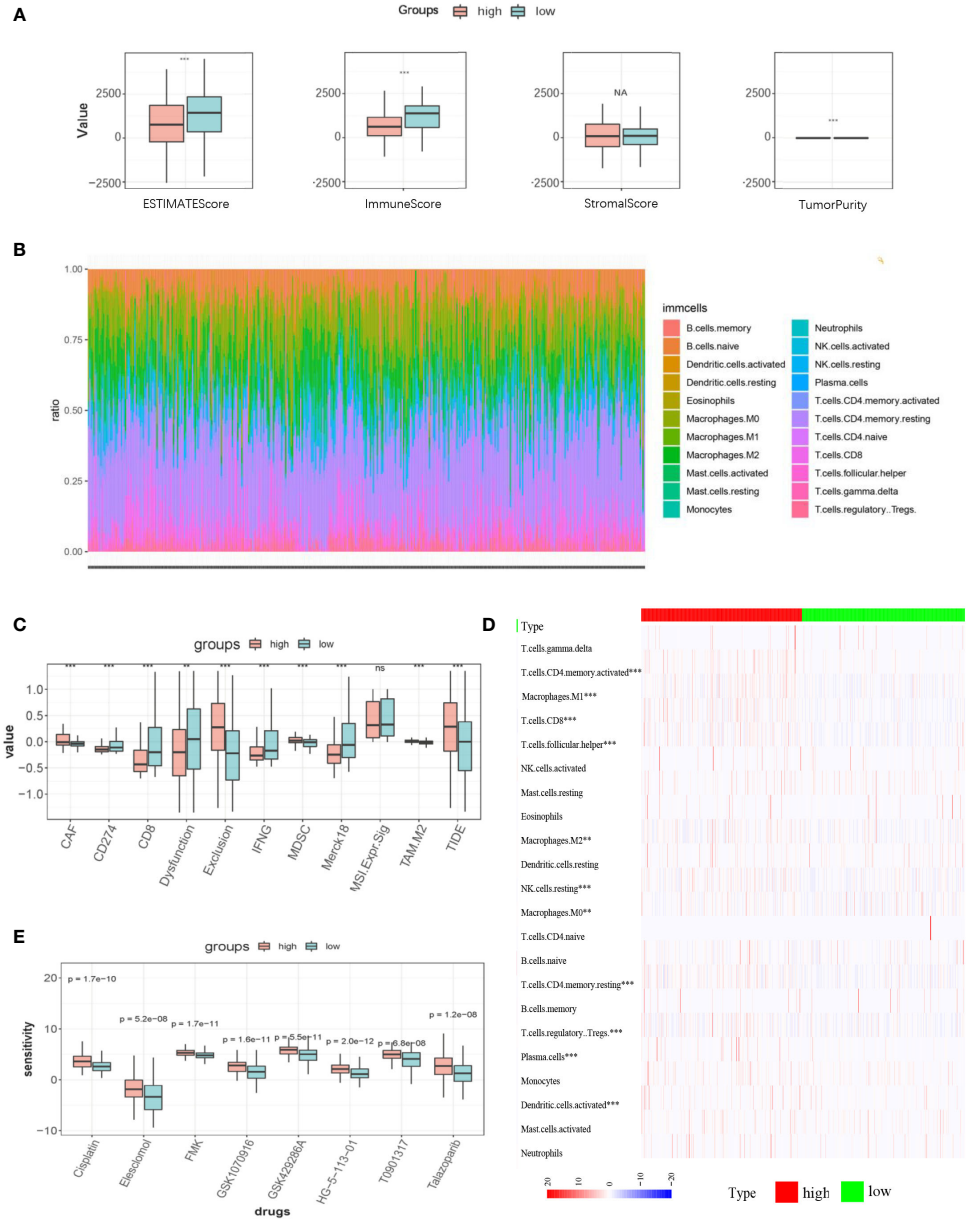
TIME characteristics, predictive immunotherapy efficacy, and chemosensitivity in high- and low-LI groups. **(A)** ESTIMATEScore, ImmuneScore, StromalScore, and tumor purity of the high- and low-LI groups. **(B)** Stacked graph demonstrating the abundance and distribution of the infiltrating immune cell in each sample. **(C)** Heatmap demonstrating the statistically significant infiltrating immune cells in high- and low-LI groups. **(D-E)** Box plots demonstrating the results of TIDE prediction of high- and low-LI groups **(D)** and the comparisons of chemosensitivity for each drug **(E)**. Ns, p≥0.05; **, p<0.01; ***, p < 0.001.

The R package “ESTIMATE” was used to elucidate the general appearance of TIME quantitatively, through which we found that except for the stromal score, the immune score, ESTIMATE score, and tumor purity were statistically significant and that higher immune and ESTIMATE scores were observed in the low-LI group than that of the high LI group (**Figure 6A**). Therefore, we also explored the abundance of infiltrating immune cells in each patient. As a result, although certain fluctuation was observed in their distribution, conclusively, it was thought that the immune cell infiltration was obvious regarding both LI groups (**Figure 6B**). To show the difference between the high- and low-LI groups, we revealed an informative but visually clear comparison as a heatmap, through which specific immune cells such as CD4 memory T cells, CD8 T cells, follicular T helper cells, regulatory T cells, Macrophages M0, M1, and M2, etc. were with statistical significance (**Figure 6C**). Furthermore, the TIDE algorithm was applied to predict the immunotherapy efficacy. Subsequently, we confirmed the close association of CD8 T cells, the main force against tumor malignancy, with LI groups (**Figure 6D**). Meanwhile, according to the explanation of the developers, higher TIDE scores are usually accompanied by poor immunotherapy efficacy. Through the results, it was found that the higher LI group corresponded with lower TIDE scores, hindering a potential advantage for the higher LI population to receive immunotherapy. Finally, we screened the possible drugs targeting LI genes from the authorized database Cancer Genome Project (CGP). Remarkable differences in the IC50 values of 8 drugs, including Cisplatin, Elesclomol, FMK, GSK1070916, GSK429286A, HG−5−113−01, T0901317, and Talazoparib were noticed between high- and low-LI groups (**Figure 6E**). Of note that the lower LI groups demonstrated reduced IC-50 values for all 8 drugs, which suggested that patients in the lower LI group were more sensitive to chemotherapy.

### Aberrant overexpression of IFI30 in gastric cancer impacts on tumor cell viability

**Figure 7:**
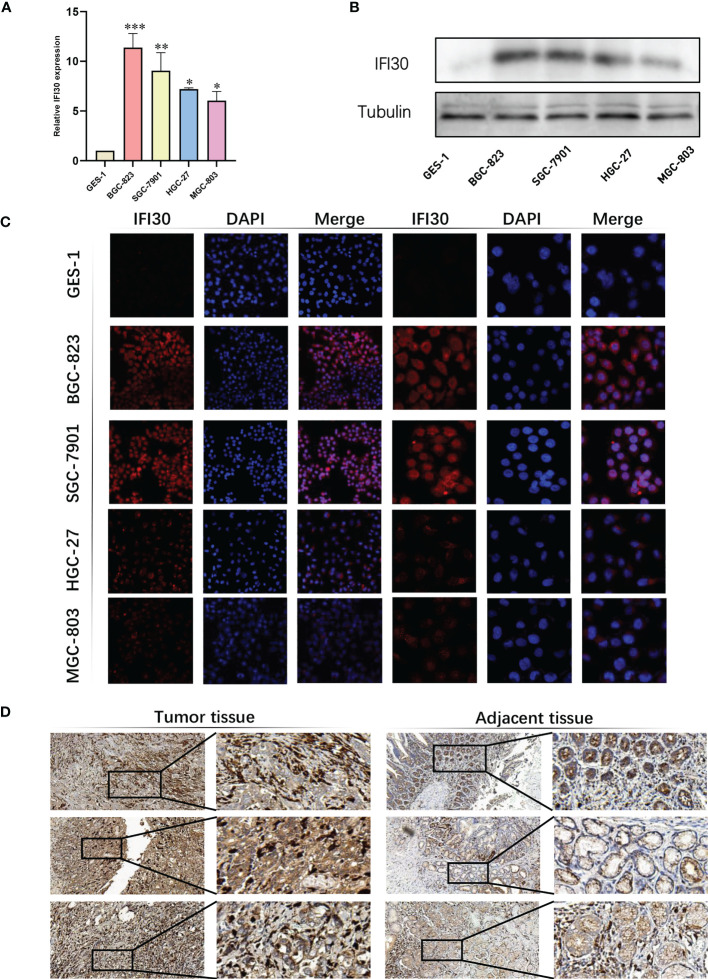
Validation of IFI30 as a potential diagnostic biomarker in GC. **(A)** Results of RT-qPCR of IFI30 in GES-1, BGC-823, SGC-7901, HGC-27, and MGC-803 cell lines, respectively. **(B)** Results of Western blot in GC cell lines. **(C)** IFS slides in 100X and 400X magnification demonstrated the expressional abundance of IFI30 in GC cell lines. **(D)** Immunohistochemical staining of the 3 pairs of real patients’ samples together with their para-cancerous tissues(20X). *p< 0.05; **, p<0.01; ***, p < 0.001.

**Figure 8:**
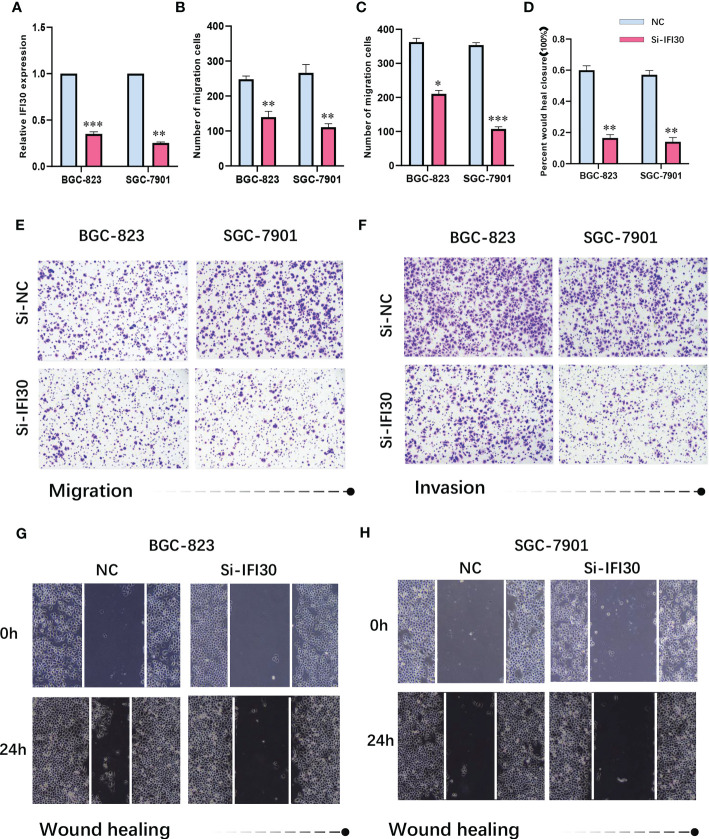
**(A)** siRNA-IFI30 efficiently depresses the expression of IFI30. **(B)** Relative cell migration number of migration assay. **(C)** Relative cell invasion numbers in the invasion assay. **(D)** Relative scratch healing area of BGC-823 and SGC-7901. **(E)** Migration assay after reduction of IFI30 expression in BGC-823 and SGC-7901. **(F)** Invasion assay after reducing the expression of IFI30 in BGC-823 and SGC-7901. **(G, H)** Wound healing assay after reducing the expression of IFI30 in BGC-823 and SGC-7901. *p< 0.05; **, p<0.01; ***, p < 0.001.

To further confirm the high diagnostic efficacy of the model we constructed, after excluding the relatively well-studied genes in GC in previous literature, we decided to focus on IFI30 as a wet-lab validation. We examined the expression levels of the mRNA and protein in normal gastric mucosal epithelial tissues and four gastric cancer cell lines. At the transcriptional level, the results of RT-qPCR revealed that IFI30 was significantly higher expressed in tumorous cell lines than that in GSL-1 (**Figure 7A**). This was further verified by the results of Western Blot at the protein level (**Figure 7B**). Overall, the trend was consistent for both validations, where BGC-823 exhibited the highest IFI30 expression levels, followed by SGC-7901, HGC-27, and MGC-803. As a supplementary, such conclusions were also supported by our immunofluorescence assays (**Figure 7C**). The immunohistochemical staining of the 3 pairs of real patients’ samples together with their paracancerous tissues likewise supported the aforementioned conclusions (**Figure 7D**). Further to our study, the siRNA for IFI30 was designed and PCR assays verified that the current siRNA was able to attenuate the expression of IFI30 significantly (**Figure 8A**). When the expression of IFI30 in GC cells was attenuated, a significant decrease in migration and invasive ability was observed, suggesting that the genes involved in the model construction are a major risk factor for gastric cancer, regardless of the model itself (**Figures 8B-H**).

## Discussion

What is expected to change the plight of gastric cancer treatment is that early diagnosis of gastric cancer gives patients a 90% chance of survival, while advanced gastric cancer has less than a third chance of survival due to significant heterogeneity (22). Therefore, the early diagnosis of gastric cancer is still the field of efforts of many scholars, even more critical (22, 23). On the other hand, targeting lysosomes showed immense potential in the treatment of diseases ranging from malignancies, although current therapeutic tools are limited by the precise targeting of lysosomes and subsequent modulation measures (9). Indeed, both the process of autophagy itself and the autophagic lysosomal pathway has been the subject of a flurry of research in human cancers due to their potential as a treasure trove for deciphering diverse diseases (24). Autophagy represents a regulatory mechanism to sustain cellular dynamic homeostasis by degrading cellular components eliminated by a series of stresses like senescence or damaging (25, 26). The appropriate conduct of the autophagic system relies on the degradative capacity of lysosomes, a process that carries out in response to tolerance to cellular stress induced by starvation or proteotoxic aggregates (25, 27). When cells themselves undergo growth imbalance, or straightforwardly, cancer, the autophagy-lysosome pathway adapts in response to abnormal stress signals in the tumor microenvironment, thereby differentially affecting tumor progression, a process that involves key hallmarks such as immune infiltration and tumor metabolism and could either suppress tumors or contribute to cancer (26).

For gastric cancer, Tan et al. previously used analysis of genome-wide association study data to confirm that genetic variation in genes across the autophagic lysosomal pathway may be significantly associated with susceptibility to gastric cancer (24). More than data analytics, Kuang et al. made their attempts to develop a new lysosomal-targeted therapeutic agent for gastric cancer, significantly highlighting such possibilities (28). Meanwhile, machine learning methods are nowadays mainly applied to the processing of medical images of gastric cancer, including endoscopy, radiological imaging, and pathology techniques, from which radiomics and pathomics have been derived (29, 30). However, the exploitation of artificial intelligence for genomic information to develop a more complete study on which to establish a diagnostic prediction model for gastric cancer would be complementary to macroscopic features and would provide new personalized medicine opportunity for patients with gastric cancer.

Based on the assumptions described above, our study confirmed such a possibility. To begin with, we selected eight lysosomal-related genes for predictive model construction based on the inclusion of RMSE as a reference standard and RF algorithm for ranking, namely ADRB2, KCNE2, MYO7A, IFI30, LAMP3, TPP1, HPS4, and NEU4. The ADRB2 signaling pathway has been recognized in previous studies as being able to serve in gastric carcinogenesis and metastasis as a β- adrenergic stress activation and might involve autophagy in this process, along with being shown to act as a prognostically negative biomarker for gastric cancer (31–33). Multiple histological evidence proves that KCNE2 is expressed at lower levels in gastric cancer than in normal tissues, and its deficiency is likely to be a potential risk factor for gastric cancer (34, 35). Unlike the former two, the biological function of LAMP3 (CD208) is mainly through its influence on the tumor microenvironment of gastric cancer. In an earlier study, Ishigami et al. noted that LAMP3 could be considered a marker of mature dendritic cells owing to its specific expression upon activation of human dendritic cells, speculating that the degree of infiltration of CD208-positive cells was negatively correlated with surgical outcome in patients undergoing radical gastric cancer surgery (36). This was detailed by Sun et al. as the involvement of LAMP3+ DC in mediating T-cell activity and with the ability to form aggregation sites for cell-to-cell interactions in the gastric cancer tumor microenvironment, from which they draw, and emphasized the possibility of targeting LAMP3 for GC (36, 37). Besides, TPP1 was also demonstrated to act as a biomarker for gastric cancer during its progression (38). IFI30, MYO7A, HPS4, and NEU4 have not been mentioned in studies on the background of gastric cancer, and we speculate that these four genes possess sufficient potential to influence the progression of gastric cancer, and further exploration could potentially facilitate the betterment of the current situation in gastric cancer research and clinical application. In fact, our experimental validation of IFI30, the most contributing gene, did confirm such claims to a certain extent. Previous studies similarly demonstrated the ability of IFI30 either to affect the redox of cells, leading to the regulation of autophagy, cell activation and proliferation, or to modulate the T-cell tolerance and thus bridge the potentially arising autoimmunity (39). In recent years, multitudes of scholars extended this to the direction of tumor immunity, thus revealing the great potential of IFI30 in the tumor immune microenvironment. By way of example, in melanoma, IFI30 could boost the processing and presentation of tumor antigens, TRP1 and TRP2, resulting in enhanced anti-tumor T-cell responses and ultimately higher patient survival (40–42). Same potential was observed in DLBC, BRCA, COAD, GBM and elsewhere (42, 43).

Furthermore, we compared current mainstream machine learning algorithms based on the eight genes screened in an attempt to discover the optimal predictive model. Taking accuracy, precision, recall, and F1 measurements into account, a preliminary determination of our study was carried out by means of applying the extra tree and random forest algorithms, incorporating the ROC-AUC value as a consideration, we concluded that the Extra Tree model, constructed based on lysosomal genes, would be the optimal option for diagnosis, which was further supplementally evidenced by the DCA curves and learning curves. Thereafter, we hypothesized that, together with gastroscopy, which is currently the main screening tool for early gastric cancer, the Extra Tree model would be applied to provide complementary screening and, in line with the predictions of the model, a comprehensive assessment of risk factors and actively control them to reduce or delay the occurrence and progression of the disease, or to promptly initiate secondary and tertiary prevention to counteract the deterioration of the disease (44–46).

To further confirm the feasibility of these observations, samples were scored and grouped using ssGSEA, with the three dominant enrichment analyses conducted on the different groups focusing primarily on the two key terms, secretion, and keratinization. We speculated on this from a pathological and morphological point of view, given that the cancerous tissue itself allows for deregulated growth beyond the normal structure in terms of hallmark, and that, in relation to the structure of the gastro-glandular body, it is conceivable that gastric glandular cells with a cancerous tendency would exhibit abnormalities in both gastric acid secretion and keratinization of their cells, which is somewhat consistent with our existing study (47). Additionally, we tried to pursue immunotherapy as a direction to get a response (48). Firstly, we assessed the abundance of immune cells in two subgroups based on LI and revealed that immune infiltrating cells, which perform an integral role in the tumor microenvironment, differed significantly between the two subgroups, which seems to confirm our suspicions, as current studies proved that differences in the immune microenvironment could be an essential contributor to drug resistance or sensitivity to the immunotherapy (49–51). We then used the TIDE algorithm to predict the effect of immunotherapy based on our current model, with the results implying that a higher LI group corresponds to a lower TIDE score, thus giving an indication that patients in this subgroup might potentially enjoy an advantage in receiving immunotherapy, which could largely optimize current gastric cancer treatment regimens. Not only that, the available data prove that the clinical efficacy of single therapies is not sufficiently superior to the combination therapies currently being explored, mainly combined chemotherapy, targeted therapy and radiotherapy, for which escalating therapeutic combinations would offer a personalized therapeutic weapon for gastric cancer patients (50, 52). Prediction of drug targets based on transcriptomic data is now a common tool (53). Taking this as a starting point, we screened possible drugs targeting the LI gene. 8 drugs such as cisplatin, eletriptanil, FMK, GSK1070916, GSK429286A, HG-5-113-01, T0901317 and talazopanib caught our attention and the results implied that patients in the lower LI group were more sensitive to chemotherapy with the above drugs, which might possibly contribute some help to individualized treatment of GC going forward.

Overall, our study essentially focused on the potential application of lysosomal-related genes in GC itself by integrating around 20 mainstream machine learning algorithms to construct an AI-based diagnostic predictor, whose development of the lysosomal index (LI) enables excellent immune assessment of patients as well as drug prediction, which would render a unique benefit to clinically intelligent adjunctive therapeutic approaches for GC patients, thereby facilitating the application of personalized management regimens.

## Data availability statement

The raw data supporting the conclusions of this article will be made available by the authors, without undue reservation.

## Ethics statement

Data retrieved from the TCGA database was collected from patients who provided informed consent based on guidelines laid out by the TCGA Ethics, Law, and Policy Group. The procedures used in this study adhere to the tenets of the Declaration of Helsinki and approval was obtained from the ethics committee of the Affiliated Hospital of Jiangsu University.

## Author contributions

QW, YL, ZZL, and YT designed the present study. QW, YL, ZZL, and YT prepared the figure and drafted this manuscript. XH, SZ, LW, and YL provided professional guidance in pathology. XH, SZ, LW, and YL are responsible for data curation and preprocessing. WL, HX, and ZZL are in charge for visualization. WL, HX, and ZZL enhanced the figures. WL, HX, and ZZL polished the language. ZRL and MX edited and revised the manuscript. ZRL and MX supervised the project. MX is responsible for funding acquisition. All authors contributed to the article and approved the submitted version.
