# Supplementary figures and images for: Establishment of a novel lysosomal signature for the diagnosis of gastric cancer with in-vitro and in-situ validation
Source: Front Immunol. 2023 May 5;14:1182277. doi: 10.3389/fimmu.2023.1182277 (PMC10196375; doi:10.3389/fimmu.2023.1182277)

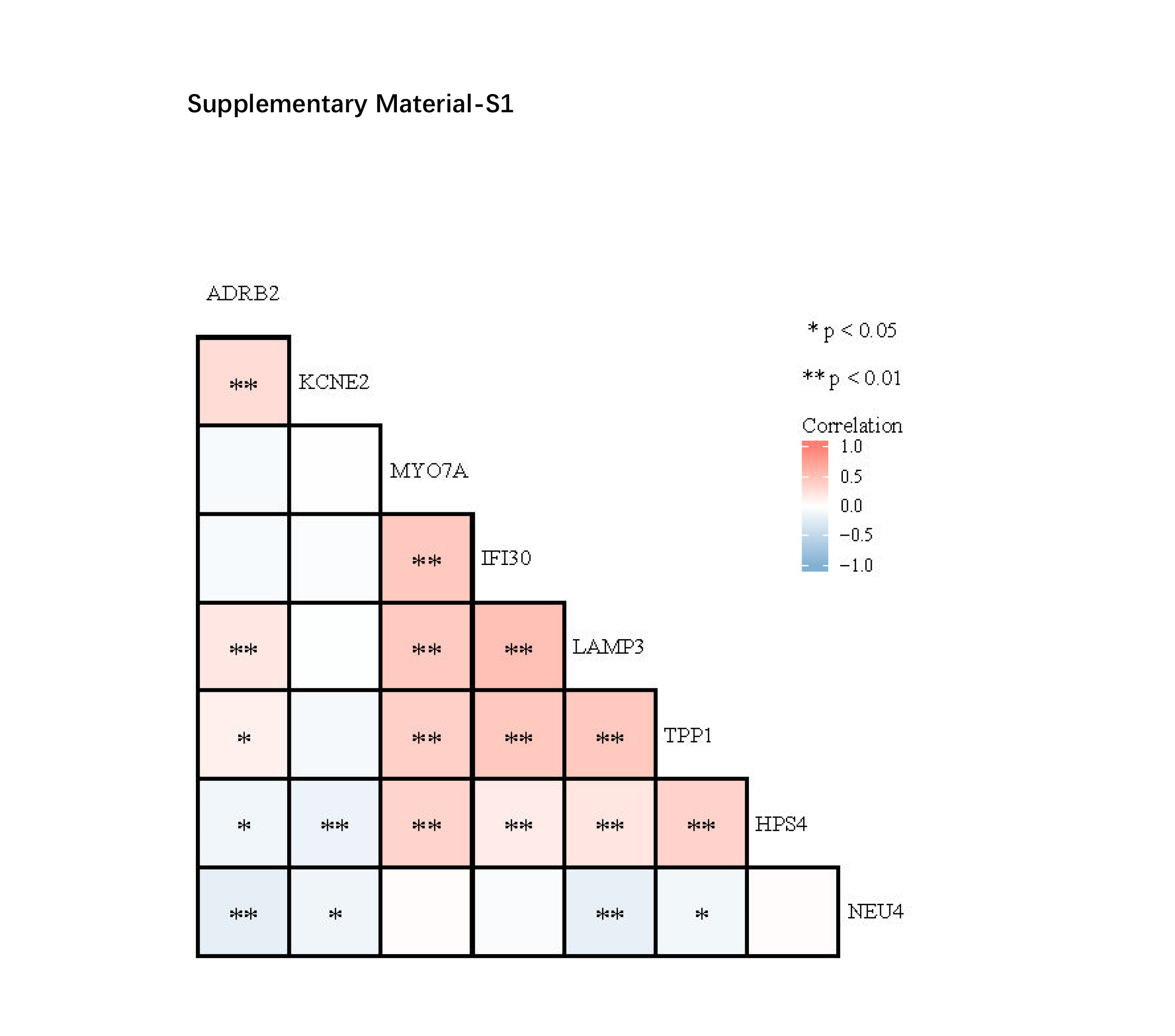

Supplement: Supplementary file 1 [file Image_1.tif]
